# Retracted: Therapeutic Management of the Hallux Rigidus

**DOI:** 10.1155/2014/130543

**Published:** 2014-05-21

**Authors:** Rehabilitation Research and Practice

The paper titled “Therapeutic Management of the Hallux Rigidus” [1], published in Rehabilitation Research and Practice, has been retracted as it was found to contain substantial flaws in its scientific methodology.
